# Quality of Documentation in Paediatric Supracondylar Fractures: A Quality Improvement Project

**DOI:** 10.7759/cureus.31431

**Published:** 2022-11-12

**Authors:** Vusumuzi Sibanda, Marjan Raad, Peter I Legg, Alex Chipperfield, Matthew C Oliver

**Affiliations:** 1 Trauma and Orthopaedics, William Harvey Hospital, Ashford, GBR; 2 Trauma and Orthopaedics, Darent Valley Hospital, Dartford, GBR

**Keywords:** fracture in a child, humeral fracture, elbow trauma, boast guidelines, supracondylar fractures

## Abstract

Introduction

Supracondylar fractures are the most common elbow fractures in children. Their documentation and management must be done fully and correctly. This Quality Improvement Project (QIP) assessed the quality of documentation for paediatric supracondylar fractures admitted, in accordance with the British Orthopaedic Association Standards for Trauma (BOAST).

Methods

We present a case series of supracondylar fractures presenting to a single UK-based district general hospital from January 2018 - October 2021. We performed a quality improvement intervention starting in November 2020. The retrospective data prior to intervention (January 2018-November 2020) were deemed “pre-intervention”. Prospective “post-intervention” data were collected from April to October 2021. After “pre-intervention” data analysis, an intervention in the form of a documentation proforma was developed and multidisciplinary teaching sessions were delivered. Post-intervention prospective data collection followed from April - October 2021.

Results

There were 48 and 26 patients in cycles one and two, respectively. The mean age was 6.4 (SD 3.5) and 6.5 (SD 2.7) years, respectively; 42/48 in cycle one and all 26/26 in cycle two required operative management. The mean time to surgery was 1.3 and 0.96 days, respectively. Post-intervention, cycle two showed a reduction in patients with “no neurovascular documentation” pre-reduction (17% vs 12%) and an increase in patients documented as having "neurovascular status intact" (NVI) (44% vs 69%). In post-operative documentation, there was an improvement in documentation with 73% of patients having a post-operative neurovascular assessment documented, versus 50% in the pre-intervention cohort.

Conclusion

This QIP provided some early improvement in the documentation but with room for future progress as the project continues. It showed proformas can be an effective tool in implementing positive change. It also highlights the need for continuous clinical education across the multidisciplinary teams managing trauma.

## Introduction

Supracondylar fractures of the distal humerus are one the most common fractures in children. They are the most common elbow fracture in children and are the most common fractures in children under seven years of age [1]. Recent studies have not shown any significant difference in the distribution of fractures based on gender, however, girls tend to sustain supracondylar fractures at a younger age than boys [2,3].

Most supracondylar fractures occur following a fall onto an outstretched hand [4]. The non-dominant extremity is more commonly affected. In children under three years, these fractures may occur in low-level falls (from a height of less than 3 feet such as a fall from a bed or couch). In the older child, most fractures occur from higher falls from playground equipment such as monkey bars, swings, or other high-energy mechanisms [5].

The Gartland Classification classifies supracondylar fractures into three groups based on the degree of displacement: type I, non-displaced; type II, moderately displaced with an intact posterior cortex, and type III, severely displaced with no cortical contact [6]. This classification helped improve and standardise the treatment of these injuries. Over the years, there have been several modifications to the classification to keep up with new evidence [7]. Non-operative treatments range from immobilisation in above-elbow plaster of Paris (POP) to the more seldom used skin traction methods, such as Dunlop’s traction. Surgical options include closed or open reduction and Kirschner-wire (K-wire) fixation. In general, type I fractures are treated conservatively with an above-elbow POP. Management of type II fractures remains controversial and the modification of the Gartland classification to include type IIa and IIb pertaining to the presence of rotational deformity is often used to help guide treatment decisions [8]. Type III fractures are typically treated operatively with closed reduction and K-wire fixation [9].

Suboptimal treatment of supracondylar fractures can result in complications that can adversely impact the child's quality of life and long-term function. Complications can be a result of the initial trauma, such as neurovascular injury and compartment syndrome. Complications can also occur secondary to treatment. These include loss of reduction, neurovascular compromise, compartment syndrome, infection, malunion with angular deformity, stiffness, ossifying myositis, and avascular necrosis of the trochlea. Canales-Zamora et al. in 2020 reviewed 277 patients and found the following percentages for complications: cubitus varus 3.97%, neurological lesions prior to surgical treatment 1.44%, early neurological complications to treatment 1.44%, infections of K-wires 0.72%, cubitus valgus 0.72%, and loss of mobility 0.36% [10,11].

Given the prevalence of supracondylar fractures and the potential complications that can occur, it is crucial to ensure they are assessed and treated appropriately from the time of presentation. To enable standardised assessment of these injuries, the British Orthopaedic Association published the British Orthopaedic Association Standards for Trauma (BOAST) guidelines for the management of supracondylar Fractures in children [12].

This Quality Improvement Project (QIP) aimed to assess if the quality of documentation of paediatric supracondylar fractures in our Trauma Unit was compliant with the BOAST guidelines. After the initial audit, we implemented an assessment proforma, multidisciplinary teaching including all members of the emergency department and trauma team, and then performed a second cycle to assess the change in practice.

## Materials and methods

Local ethical approval was gained from the Clinical Audit Department in the East Kent Hospitals University NHS Foundation Trust, and study approval was obtained (approval number: SA/22/21-22) prior to the commencement of the QIP. A case series of paediatric supracondylar fractures were recorded at a single UK-based district general hospital functioning as a trauma unit without the involvement of tertiary paediatric-orthopaedic surgical services.

Retrospective and prospective data were collected via local institutions' contemporaneous trauma database and picture archive and communication system (PACS). Parametric and non-parametric data were analysed using student’s t-test and Pearson’s chi-squared test, respectively. A confidence interval of 95% was selected and p<0.05 was deemed to be statistically significant. Descriptive statistics were described as mean +/- standard deviations. Categorical data was stated as number (n) and percentage (%).

First cycle

We conducted a retrospective review of patients treated in our NHS Trust for paediatric supracondylar fractures from January 2018 to November 2020.

With the help of the Health Records department, we identified all the patients treated over the specified period. We reviewed the operation notes of patients who had undergone surgical intervention and the outpatient clinic letters for all the patients. From these records, we obtained information relating to whether there had been complete neurovascular documentation at the time of presentation to the accident and emergency (A&E) department, after reduction or application of cast and after surgical intervention (if applicable) according to the BOAST guidelines. Neurological assessment was considered complete when both sensory and motor function of the median, anterior interosseous nerve (AIN), ulnar and radial nerves were individually documented. The vascular assessment was considered complete when the radial pulse and capillary refill time were individually documented.

We obtained information on the patient's age, gender, Gartland classification of fracture, mechanism of injury, the treatment given, time to surgery (if applicable) and time of follow-up. Time to follow-up was defined as the time from initial contact to final follow-up or discharge in the outpatient clinic.

**Figure 1:**
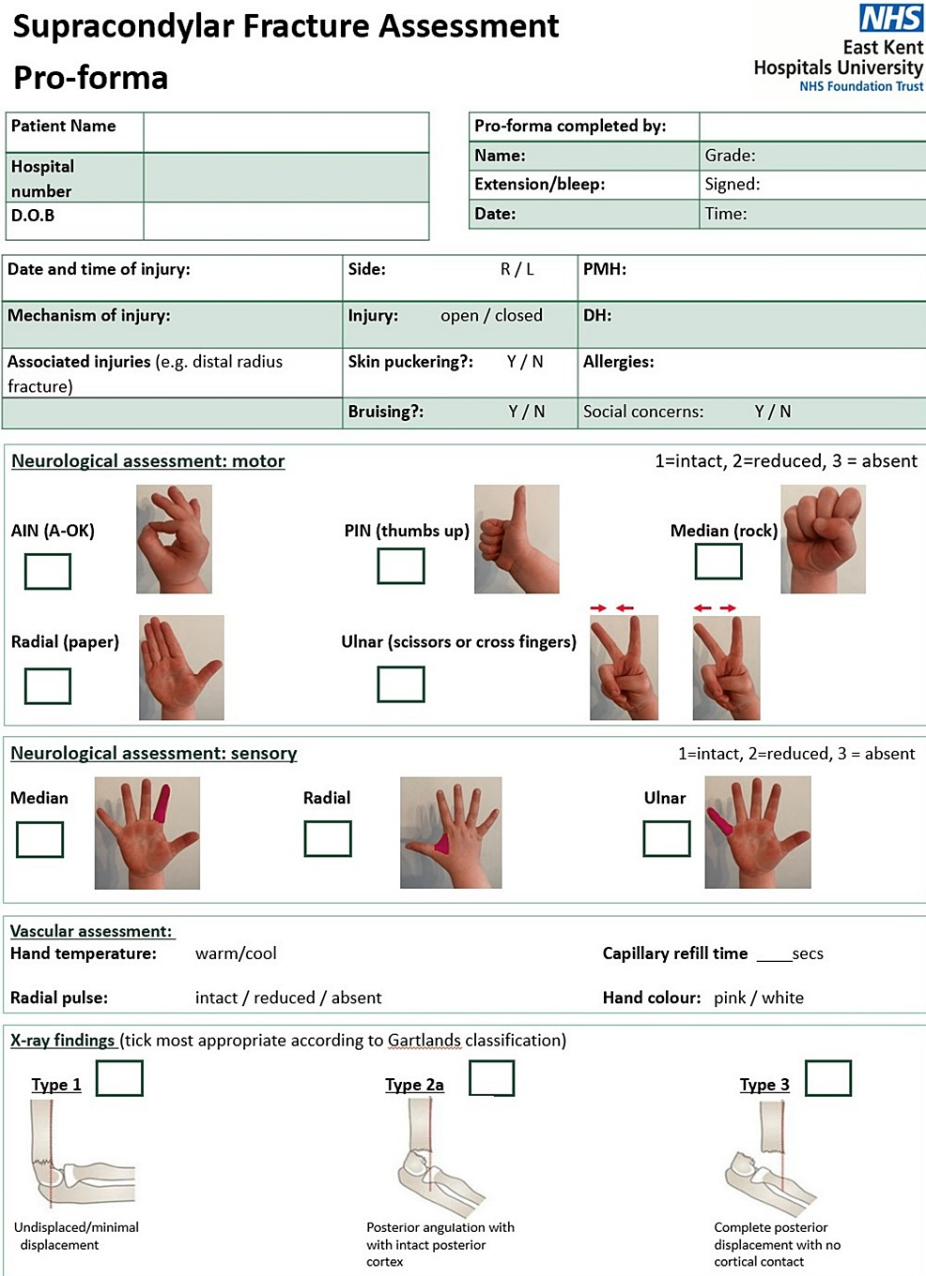
Supracondylar Fracture Assessment Documentation Proforma Page 1

**Figure 2:**
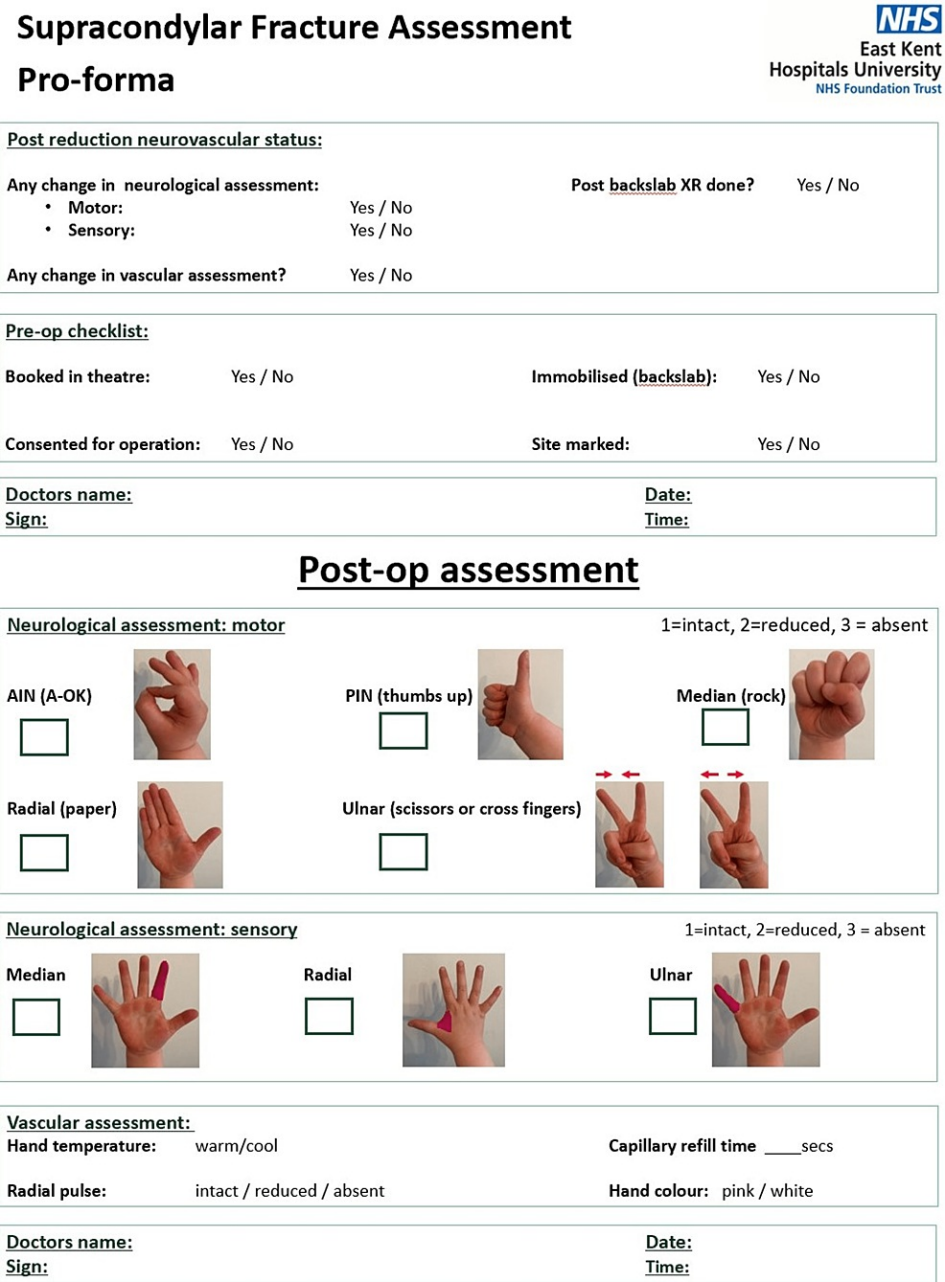
Supracondylar Fracture Assessment Documentation Proforma Page 2

After the initial audit period, we analysed the data and presented the findings in the departmental audit meeting. We recommended and adopted the following recommendations:

1. We created a supracondylar fracture assessment proforma to aid in the clerking and documentation pre and post-intervention (Figures 1-2). This proforma was made available in both paper and electronic form.

2. Multidisciplinary teaching including all members of the emergency department and trauma team. 

Second cycle

We prospectively reviewed patients treated for supracondylar fractures from April to October 2021. Information was obtained from the same sources as in the first cycle. Data collected was on documentation of neurovascular status pre and post-intervention, patient age and gender, Gartland classification, mechanism of injury, the treatment offered, time to surgery (if applicable), and time to follow up.

## Results

First cycle

**Figure 3:**
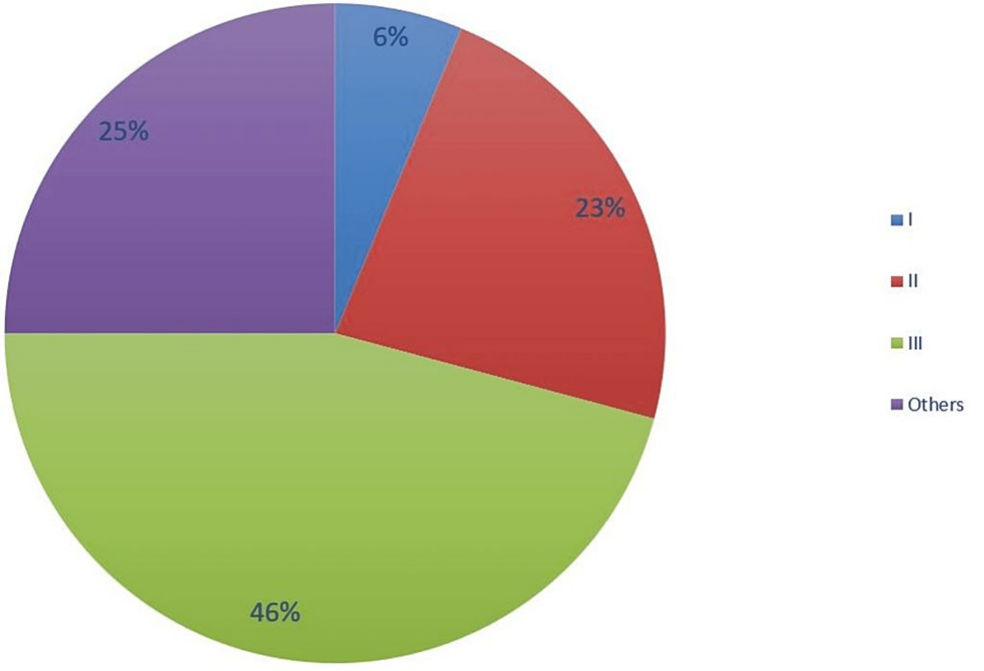
Gartland Fracture Types Cycle 1

A total of 48 patients were included in the first cycle. Of these, 27 (56%) were male, and 21 (44%) were female. The mean age of the patients was 6.4 years (SD 3.5). Of the 48, 36 had true supracondylar fractures, and 12 (25%) had medial and lateral epicondyle fractures. Of the 36 supracondylar fractures, three (6%) had Gartland type I fractures, 11 (23%) had type II fractures, and 22 (46%) had type III fractures (Figure 3).

Forty-two (87.5%) of the 48 patients had operative treatment for their fractures. Thirty-sex (85.7%) had closed reduction and percutaneous pinning, four (9.5%) had open reduction, and internal fixation with cannulated screws, and two (4.8%) had closed reduction under anaesthesia and application cast. Mean time to surgery was 1.3 days and the average follow-up period was 74 days.

Only 19 (39.6%) of the patients had full neurovascular documentation at the time of first review in A&E, with 21 (43.7%) documented as "neurovascular intact" and eight (16.7%) having no neurovascular documentation at all. After the application of a backslab in A&E, 16 (33.3%) of the patients had full neurovascular status documentation, eight (16.7%) were documented as "neurovascular intact", and 24 (50%) had no documentation. Of the 42 patients who had surgery, 16 (38%) had full neurovascular status documentation postoperatively, five (12%) were documented as "neurovascular intact" and 21 (50%) had no documentation (Table 1).

**Table 1:** Documentation of Neurovascular (NV) Status Cycle 1 NVI: Neurovascular intact

	Full NV Status Documentation pre-backslab?	Full NV Status Documentation post backslab?	Full NV status Documentation post operatively?
Yes	19	16	16
No	8	24	21
Documented as “NVI”	21	8	5

Second cycle

**Figure 4:**
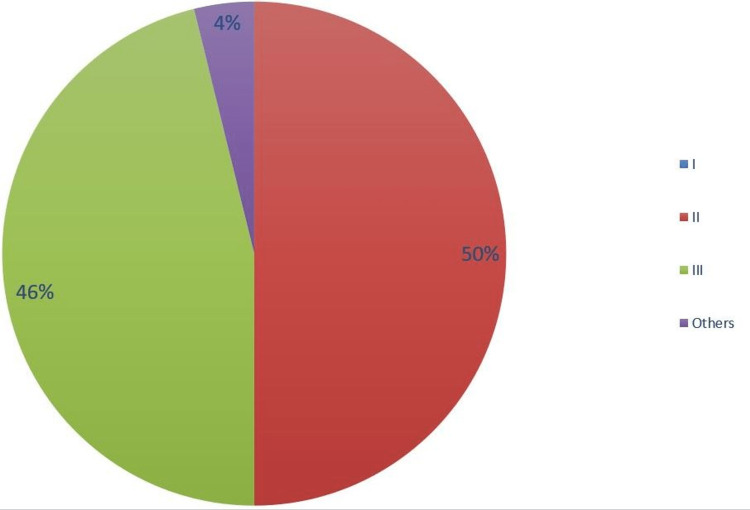
Gartland Fracture Types Cycle 2

In the second audit cycle, 26 patients were included, 17 (65%) male and nine (35%) female. The mean age was 6.5 years (SD 2.7). Thirteen (50%) patients had Gartland type II fractures, 12 (46%) had Gartland type III and one (4%) had an intra-articular T-type fracture (Figure 4). All 26 patients had operative management, with 24 (92%) having closed reduction and percutaneous pinning, one (4%) had closed reduction under anaesthesia and application of backslab, and one (4%) had open reduction and internal fixation with plates and screws. Mean time to surgery was 0.96 days and the average time to follow-up was 51 days.

Five (19.2%) of the patients had full neurovascular documentation at the time of first review in A&E, with 18 (69.2%) documented as "neurovascular intact" and three (11.6%) had no neurovascular documentation. After application of a backslab in A&E, nine (34.6%) had full neurovascular status documentation, four (15.4%) were documented as "neurovascular intact", and 13 (50%) had no documentation. Postoperatively, 10 (38.5%) had full neurovascular status documentation, nine (34.6%) were documented as “neurovascular intact” and seven (26.9%) had no documentation (Table 2).

**Table 2:** Documentation of Neurovascular (NV) Status Cycle 2 NVI: Neurovascular Intact

	Full NV Status Documentation pre-backslab?	Full NV Status Documentation post backslab?	Full NV status Documentation post operatively?
Yes	5	9	10
No	3	13	7
Documented as “NVI”	18	4	9

**Figure 5:**
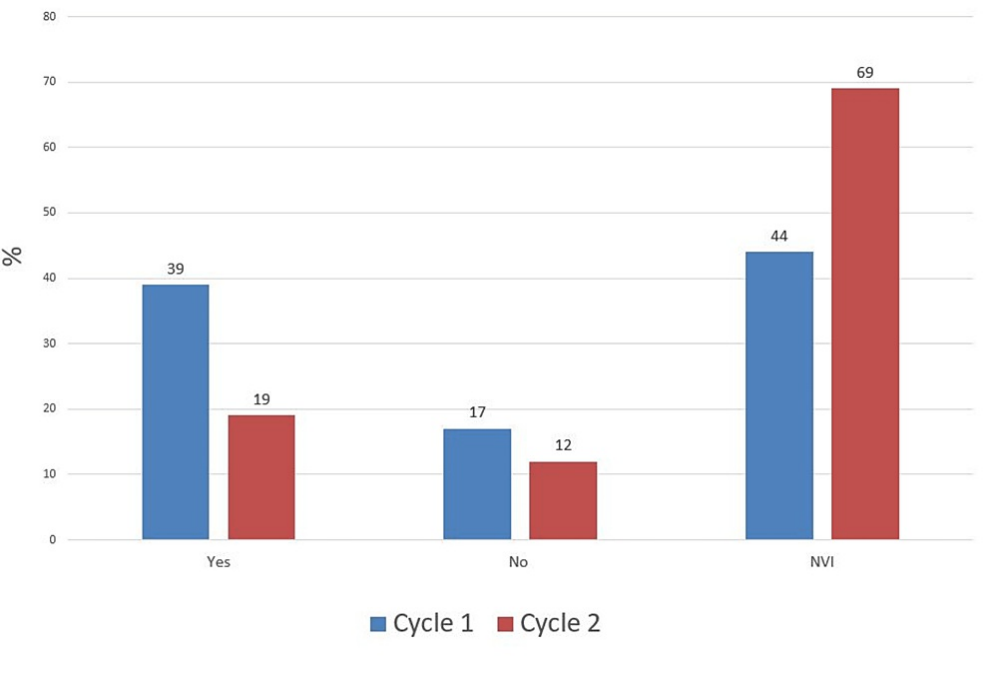
Pre-Reduction Neurovascular (NV) Status Documentation NVI: Neurovascular Intact

**Figure 6:**
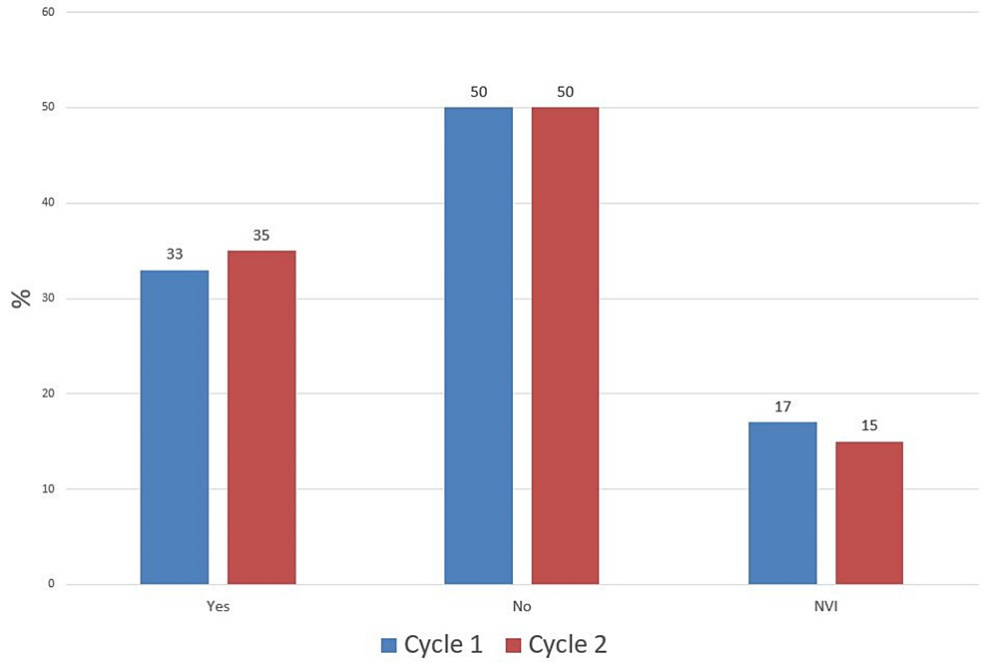
Post Application of Backslab Neurovascular (NV) Status Documentation NVI: Neurovascular Intact

**Figure 7:**
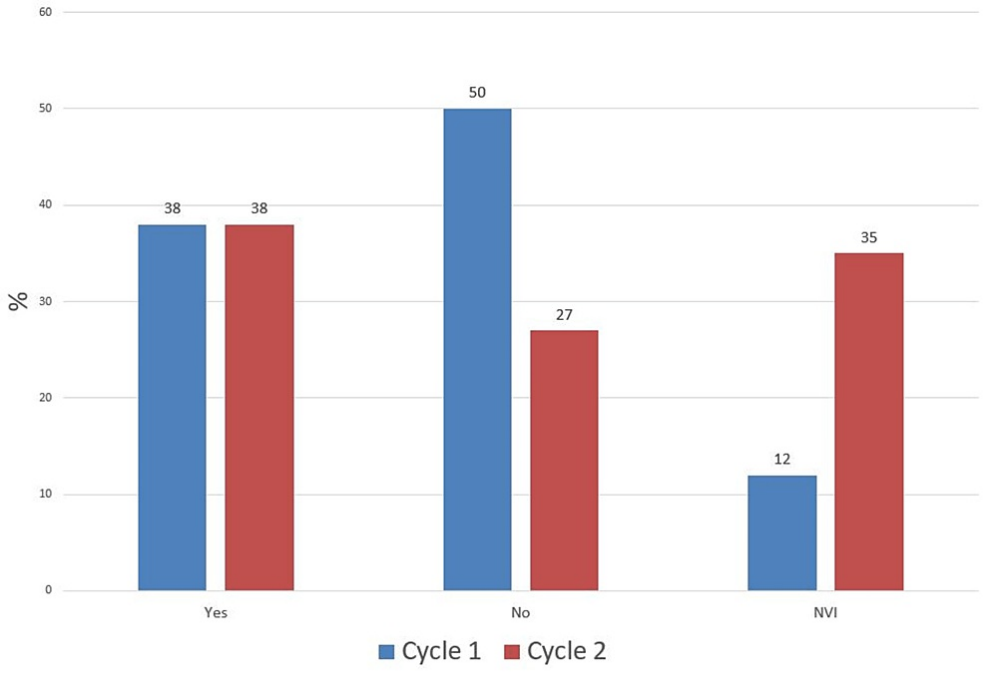
Post-operative Neurovascular (NV) Status Documentation NVI: Neurovascular Intact

In comparison, pre-reduction, there was a decrease in the number of patients who had no neurovascular status documentation in the second cycle (12% vs 17%) and an increase in those who were documented as "neurovascular intact" (69% vs 44%) with an associated drop in patients with full neurovascular documentation (19% vs 39%) (Figure 5). After application of backslab, the proportion of patients with no NV status documentation remained at 50% while there was an increase in the percentage of those with full NV documentation (35% vs 33%) and a drop in those documented as NVI (15% vs 17%) (Figure 6). Postoperatively in the second cycle, the proportion of patients with complete neurovascular documentation remained the same (38% vs 38%). However, there was a significant drop in patients with no neurovascular documentation (27% vs 55%) and an associated increase in patients documented as "neurovascular intact" (35% vs 12%) (Figure 7).

## Discussion

In our cohort of patients assessed in this QIP, the average age of the patients was 6.4 and 6.5 years in the first and second cycles, respectively. There was also a predominance of these injuries in boys (56% and 65%). These findings are in keeping with the mean age and gender predominance found in other epidemiological studies [13,14].

Full documentation is an essential part of good medical care. It allows accurate passage of information from one clinician to another and ensures the patients' safety. In addition, it helps monitor for the development of complications related to the injury and monitor for signs of healing and lack thereof. Complete and correct documentation also has legal implications essential in modern medical practice [15]. In our study, a number of clinicians documented the neurovascular status of the patients as "neurovascular intact." This form of documentation is incomplete and inadequate as it does not fully demonstrate the function of the different nerves and vessels in the upper limb. The use of documentation proformas such as the one we introduced in our study has been shown to improve compliance with documentation. Marsh et al. in 2016 showed that the introduction of a simple guideline resulted in improved quality of documentation of neurological assessment and nerve injury detection [16].

The timing of surgery for supracondylar fractures has been a subject of debate for a long time. Some authors have advocated for operating on supracondylar fractures as soon as possible to minimise complications, especially in Gartland type III fractures [17]. However, other studies do not show the increased benefit of emergent surgical treatment of these fractures [18]. The BOAST guidelines state that surgical management, if needed, should be carried out on the day of injury and night-time operating is not necessary unless there are indications for urgent surgery. In addition, operating on supracondylar fractures at night has been shown to be associated with worse outcomes for patients. Aydoğmuş et al. showed that patients with supracondylar fractures who underwent surgery during non-working hours had poor reduction quality, while Paci et al. demonstrated that the mal-union rate was higher in patients operated at night during non-working hours [19,20]. In our cohort, most patients who required surgery had their surgery within a day of presenting to the hospital, with a mean time to surgery of 1.3 and 0.96 days in the two cycles, respectively.

The majority of the patients who underwent surgical operations in our study had closed reduction and percutaneous pinning (CRPP). All the patients who had CRPP for supracondylar fractures had at least two K wires inserted in either a converging (cross) configuration or diverging (lateral) configuration. Those who had converging (cross) wires had one wire inserted through the lateral humeral epicondyle and another through the medial epicondyle. There was no difference in outcomes in the two groups of patients in our cohort. In contrast, studies have shown that converging or cross-K wires have superior stability since they resist axial rotation. However, they carry the additional risk of iatrogenic injury to the ulnar nerve [21]. Relative risk (RR) of the ulnar nerve injury is as high as 4.3 times with crossed pins compared to lateral-entry pins, with an estimated incidence of 3.4% versus 0.7% [22]. As a result, some surgeons prefer to use lateral K wires only. Skaggs et al. showed that the lateral-only configuration has stability that is comparable to cross-wire configuration even for most unstable fractures [23].

There are some limitations to our study. Our study did not show drastic improvement in the quality of documentation over the two audit cycles. This could partly be due to the fact that documentation of the neurovascular status of patients is done by junior doctors (RMOs, SHOs, Core Trainees) and middle-grade doctors (Registrars and Clinical Fellows). Doctors in these roles tend to rotate between different hospitals and NHS Trusts as part of their training. Ensuring standards are maintained over a long period with new personnel coming can be challenging. This challenge can be overcome by ensuring that healthcare professionals involved in trauma care and other branches of medicine undergo continuous medical education. Continuous medical education allows healthcare providers to refine skills, stay current with the latest developments within their speciality, and learn effective medical management skills [24,25]. In addition, there was a migration from paper documentation to electronic (online) documentation over the study period that we conducted our audits. Some of the information might have been misplaced or lost while we were still using paper notes.

The strengths of our study, include the relatively long period which we reviewed. In the first cycle, the review period was almost three years long. Secondly, the patients that were reviewed were not only from one centre. There were multiple hospitals audited simultaneously, including three major hospitals in our NHS Trust. This ensured a wider pool of patients and reduced bias in the analysis of the results.

## Conclusions

This QIP provided some early improvement in the documentation but with room for future progress as the project continues. It showed proformas can be an effective tool in implementing positive change. It also highlights the need for continuous clinical education across the multidisciplinary team managing trauma. Our local management of supracondylar fractures in children, however, is in keeping with national guidelines. We offer the appropriate treatment to the appropriate patients at the appropriate time for these injuries.
